# Side-Alternating Vibration Training Improves Muscle Performance in a Patient with Late-Onset Pompe Disease

**DOI:** 10.1155/2009/741087

**Published:** 2009-05-25

**Authors:** Aneal Khan, Barbara Ramage, Ion Robu, Laura Benard

**Affiliations:** ^1^Medical Genetics and Pediatrics, Alberta Children's Hospital, University of Calgary, 3rd Floor, 2888 Shaganappi Trail NW, Calgary, AB, Canada T3B 6A8; ^2^Department of Pediatrics, Faculty of Medicine, University of Calgary, 2888 Shaganappi Trail NW, Calgary, AB, Canada T3B 6A8; ^3^Riddell Movement Assessment Centre, Alberta Children's Hospital, University of Calgary, 2888 Shaganappi Trail NW, Calgary, AB, Canada T3B 6A8

## Abstract

Side-alternating vibration training (SAVT) was used for 15 weeks in a patient with Late-onset Pompe disease who had never used enzyme replacement or chaperone therapy. Prior to the use of SAVT, the patient had experienced declining muscle performance and her 6-minute walk distance decreased from 210 to 155 metres in 6 months. After SAVT, her 6-minute walk distance increased 70% from 166 to 282 metres, muscle jumping power increased by 64% from 83 to 166 watts, isometric knee extensor strength increased 17% from 38 to 44 Nm, and she achieved a more normal pattern of ankle, knee, and joint kinematics and kinetics. Her functional ability measured through the Rotterdam 9-item score was unchanged at 19/36. There were no elevations in serum creatine kinase or lactate. This is the first report, to our knowledge, of a performance improvement in a patient with Pompe disease using SAVT.

## 1. Introduction

Late-Onset Pompe disease (acid alpha-glucosidase deficiency, OMIM 606800) can lead to progressive muscle weakness and loss of mobility [1]. Many patients further develop deconditioning from decreased physical activity which compounds the declining muscle function. Side-alternating vibration training (SAVT) has been used to improve postural stability, muscle strength, and overall muscle performance in healthy subjects and athletes [2–6]. To our knowledge, the use of SAVT has not been previously reported in patients with a progressive myopathy such as late-onset Pompe disease (LOPD). We report the results of 15 weeks of SAVT in a patient with LOPD and no prior use of enzyme replacement or chaperone therapy who was experiencing declining ambulatory function. We suggest that other patients with late-onset Pompe disease may also benefit from SAVT.

## 2. Case Report

Our patient was a 34-year-old woman who was diagnosed at 30 years of age with late-onset Pompe disease. Sequencing of the *GAA* gene (Dr. Nancy Carson, Children's Hospital of Eastern Ontario) showed a c.-32-13 T > G and a c.2481 + 102_2646 + 31 deletion of intron 1 and introns 17 to 18, respectively. Her T-cell alpha-glucosidase activity was <2.5 nmol/h/mg protein (controls 28.3–66.2) (Dr. Floyd Snyder, University of Calgary). She had never used enzyme replacement therapy or any other form of drug therapy targeted to acid alpha-glucosidase deficiency. She was ambulatory but walked slowly, and required a cane in the months prior to using SAVT, had difficulty ascending or descending roadside curbs, and was unable to stand from a sitting position without the use of aids. She reported chronic weakness and fatigue resulting in difficulty using stairs. Additionally, she noted an accelerated decrease in her physical abilities over the last two years with the need to reduce the number of hours she was working. She never required ventilatory support and had normal cardiac function.

Baseline investigations included the Rotterdam 9-item scale [7], 6-minute walk test (6 MWT), grip strength (Baseline Hydraulic Dynamometer, White Plains, NY, USA), isometric hamstrings and quadriceps strength (Biodex System 3 Pro, Biodex Medical Systems, Inc., NY, USA), peak lower extremity power using a force plate (Advance Mechanical Technology Inc. (AMTI), Watertown, MA, USA), custom-written software (MATLAB, MathWorks, Natick, MA, USA), lower extremity 3D kinematics and kinetics during level walking (Motion Analysis Corp, Santa Rosa, Calif, USA; AMTI, Watertown MA, USA), serum creatine kinase, urine 24 hour myoglobin, and serum electrolytes. She was started on a protocol using a side-alternating vibration platform (Vibraflex, Galileo, Home Edition, Novotec Medical, Pforzheim, Germany). Each SAVT session comprised 60 seconds vibration-on then 60 seconds vibration-off sequence (one cycle), starting with two cycles initially, progressing to four cycles by week 11 and continuing with four cycles to week 15. Initially, the vibration frequency was 5 Hz, progressing to 20 Hz by week 11 and continuing at 20 Hz to week 15. Her stance was similar for all sessions with each hallux positioned 11 cm from the vibration axis and knees and hips slightly flexed. The subject was 100% compliant with 3 sessions per week for a total of 15 weeks. Follow-up measurements were made 5 days after her last SAVT session. Mean grip strength was calculated from 3 trials using each hand separately and served as an internal control of muscle function.

## 3. Results

### 3.1. Tolerance to SAVT

The SAVT was generally well tolerated. The following symptoms were reported as a percentage of total vibration sessions: muscle discomfort (soreness, stiffness, aches) in the legs (49%), twitching while at rest (31%), cramping (2%), and fatigue (4%). Most often, these symptoms occurred the day following SAVT would be aggravated by longer days at work and caused minor discomfort but did not cause the patient to change her activity level. Other than the muscle twitching, she did not feel that these symptoms had worsened compared to her baseline and generally improved with massage therapy. 

There was no change in her weight, blood pressure, resting heart rate, serum creatine kinase, or 24 hour urine myoglobin (Table 1). Venous blood lactate measured mid-way and at the end of the trial were 1.7 mmol/L and 1.2 mmol/L, respectively, (normal).

### 3.2. Muscle Performance

**Figure 1:**
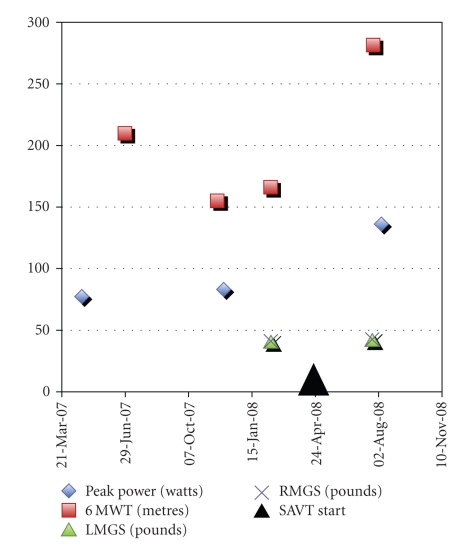
Muscle measurements before SAVT (April 21, 2007 to April 20, 2008) and after SAVT (started on April 21, 2008). Calendar date is along the *x*-axis and numerical measurements along the *y*-axis for peak power (watts), six-minute walk test distance (6 MWT; metres), left mean grip strength (LMGS; pounds), and right mean grip strength (RMGS; pounds).

After showing an initial decline in her 6-minute walk test (6 MWT) prior to commencing SAVT, the patient showed a 70% improvement in the distance traveled, from 166 to 282 metres after using SAVT (Figure 1). Peak lower extremity power using jumping mechanography improved by 64% from 83 to 136 watts. Isometric strength of the knee extensors increased 17% (from 38 to 44 Nm) while flexor strength diminished 13% (from 15 to 13 Nm). During this time period, mean grip strength was unchanged (left hand 17.7 to 18.5 kg (39.0 to 40.8 lb), right hand 18.3 to 18.8 kg (40.3 to 41.5 lb)). At week 4 of SAVT she no longer used a cane during walking. At week 14 of SAVT she was able to bend her knees without falling and able to lift her right leg off the bed. Functional abilities assessed using a 9-item Rotterdam scale were unchanged at 19/36.

Manual muscle testing (MMT) was completed by the same therapist at baseline and post-SAVT (Table 2) using a modified Medical Research Council (MRC) scale [8]. Right and left legs demonstrated the same strength, and all muscles showed improvement except knee extensors which were similar pre- and post-SAVT.

**Figure 2:**
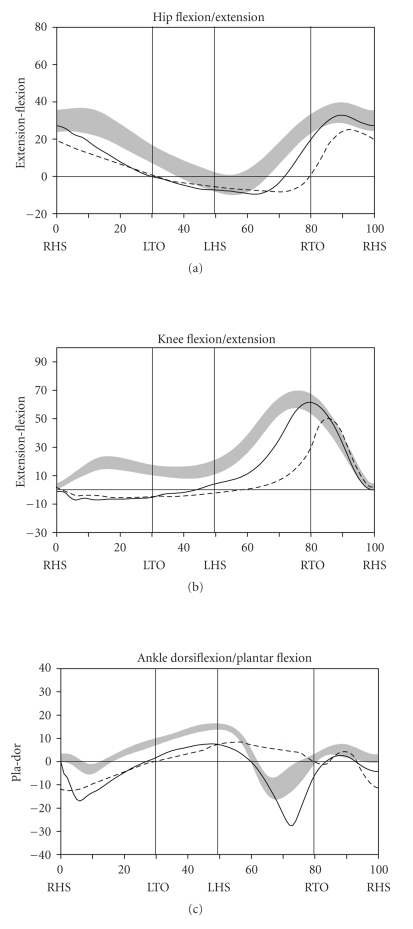
Normalized sagittal kinematics for the right hip, knee, and ankle joints for the pre- (dashed line) and post-SAVT (solid line) trials. Age-matched normal data (mean ± 1SD) are represented by the gray band. The *x*-axis is normalized stride cycle, with right heel strike (RHS) occurring at 0% of the cycle, followed by left toe-off (LTO), left heel strike (LHS), right toe-off (RTO), and RHS. The *y*-axes are the joint angles (degrees) with the zero line representing neutral joint position. The same findings were noted on the left side.

During level walking at a self-selected speed, hip, knee, and ankle joint kinematics and kinetics improved dramatically between the pre- and post-SAVT intervention, with a pattern of greater improvement distally (ankle) than proximally (hip) (Figure 2). The ankle joint demonstrated greater overall excursion, particularly early and late in the gait cycle. Knee hyperextension during stance (right heel strike to right toe-off) persisted after SAVT, but knee excursion during the swing phase (right toe-off to right heel strike) improved after intervention. Temporally, the hip improved with joint angle at heel strike and in late swing falling within the normal range. Based on the Gillette Gait Deviation Index [9], her right leg improved from a value of 73.0 to 82.8, and her left leg improved from 70.1 to 85.7, with 100 being a normal median score. 

There was no change in pulmonary status or forced vital capacity (FVC) in the patient over the course of vibration training.

## 4. Discussion

Conventional methods of improving muscle performance use resistance and endurance training. Many patients with late-onset Pompe disease report decreased motivation for exercise because of muscle weakness, generalized fatigue, shortness of breath, and a higher level of effort required. To compound the underlying myopathy, the inactivity further deteriorates muscle function through deconditioning. Even in healthy subjects, deconditioning can lead to loss of muscle bulk and performance [10, 11]. 

Vibration is a mechanical oscillation characterized by amplitude (mm) and frequency (Hz) which is applied indirectly to muscles through the joints while standing on a vibrating platform [12]. The vibration is transmitted from the platform through the muscles of the leg and hips [2–4, 13, 14]. Vibration was initially used in conjunction with exercise for training athletes to improve performance [13, 15] and as a counter-measure to reduce the impact of space-flight on muscle performance and bone health [16]. Therefore, we felt there was potential for its application in mobility-impaired patients with weakness from Pompe disease.

The 6 MWT is a standard clinical measure of walking performance [17]. The improvement in the 6 MWT in our patient is similar in distance, greater when measured in relative amounts, to the effects of strength training and cardiovascular exercise in women with fibromyalgia [18] and better than self-administered exercise programs in the elderly [19]. Muscle power using jump mechanography has a high degree of correlation with the chair rising manouvre and in patients who are not able to get up from a chair without considerable assistance, such as in our patient and other patients with Pompe disease, improvements in this activity are functionally very important [20]. Our patient showed a greater improvement in peak power using jumping mechanography compared to healthy subjects who had received a 4-month trial of SAVT [3]. Furthermore, there were dramatic improvements in gait kinetics and kinematics, particularly distally, and in a clinical index of overall gait function. Equally important, there were no functional declines in performance using the Rotterdam 9-item scale.

The results from our single case report show that SAVT can be tolerated by patients with late-onset Pompe disease and may improve muscle function and walking. We feel there is potential for using SAVT in patients with late-onset Pompe disease and impaired mobility. Whether there is a general benefit to patients and improved performance of activities of daily living will require clinical trials of longer duration involving more subjects.

## Figures and Tables

**Table 1:** Baseline and post-SAVT physical parameters and biochemistry data.

	Baseline	Post-SAVT
Weight (kg)	60	60
Resting heart rate (beats per minute)	79	84
Systolic blood pressure (mm Hg)	111	119
Diastolic blood pressure (mm Hg)	73	87
Serum creatine kinase (U/L)	1080	1060
Serum creatinine (umol/L)	41	34
24 hour urine myoglobin excretion	negative	negative

**Table 2:** Baseline and post-SAVT muscle strength.

	Baseline	Post-SAVT
Hip flexion	2+	3−
Hip extension	2−	3−
Hip abduction	2−	3−
Knee flexion	4−	4
Knee extension	3+	3+
Ankle dorsiflexion	4+	5
Ankle inversion	5	5

Modified Medical Research Council scale: 5 = completes movement through full range of motion (ROM) against gravity with maximal resistance; 4 = full ROM against gravity with moderate resistance; 3 = full ROM against gravity; 2 = full ROM with gravity eliminated. The qualifiers + and − indicate the amount of ROM the patient is able to complete within each level of resistance.
